# Interactions between Snow Chemistry, Mercury Inputs and Microbial Population Dynamics in an Arctic Snowpack

**DOI:** 10.1371/journal.pone.0079972

**Published:** 2013-11-25

**Authors:** Catherine Larose, Emmanuel Prestat, Sébastien Cecillon, Sibel Berger, Cédric Malandain, Delina Lyon, Christophe Ferrari, Dominique Schneider, Aurélien Dommergue, Timothy M. Vogel

**Affiliations:** 1 Environmental Microbial Genomics, Laboratoire Ampere, CNRS, Ecole Centrale de Lyon, Université de Lyon, Ecully, France; 2 ENOVEO, Lyon, France; 3 Université Joseph Fourier Grenoble 1/CNRS, LGGE, Saint Martin d’Hères, France; 4 Laboratoire Adaptation et Pathogénie des Microorganismes, Université Joseph Fourier Grenoble, Grenoble, France; 5 CNRS UMR 5163, Grenoble, France; International Atomic Energy Agency, Austria

## Abstract

We investigated the interactions between snowpack chemistry, mercury (Hg) contamination and microbial community structure and function in Arctic snow. Snowpack chemistry (inorganic and organic ions) including mercury (Hg) speciation was studied in samples collected during a two-month field study in a high Arctic site, Svalbard, Norway (79°N). Shifts in microbial community structure were determined by using a 16S rRNA gene phylogenetic microarray. We linked snowpack and meltwater chemistry to changes in microbial community structure by using co-inertia analyses (CIA) and explored changes in community function due to Hg contamination by q-PCR quantification of Hg-resistance genes in metagenomic samples. Based on the CIA, chemical and microbial data were linked (*p = *0.006) with bioavailable Hg (BioHg) and methylmercury (MeHg) contributing significantly to the ordination of samples. Mercury was shown to influence community function with increases in *merA* gene copy numbers at low BioHg levels. Our results show that snowpacks can be considered as dynamic habitats with microbial and chemical components responding rapidly to environmental changes.

## Introduction

The Arctic environment experiences global changes due to climate shifts, long-range transportation of contaminants and increased human activity. An important Arctic feature is the seasonal snow-cover, which extends over a third of the Earth’s land surface, covering up to 47 million km^2^ [1] and is considered to be a dynamic habitat of limited duration [2]. Snow cover influences global energy and moisture budgets, thereby, influencing climate [1]. Snow is a receptor surface and storage compartment for nutrients, soluble inorganic and organic matter, and contaminant chemicals, such as mercury (Hg), that are delivered by wet and dry deposition [3], [4]. Hg is a toxic metal whose concentration is increasing in the Arctic food chain [5] and can undergo transformations to form methylmercury (MeHg), a potent neurotoxin. Far from inert, seasonal Arctic snowpacks are chemically dynamic [6], [7] and interact with different environmental compartments such as the atmosphere, soil and meltwater-fed ecosystems.

Due to the cold conditions and the limited supply of liquid water, snow and ice have long been considered as only entrapment and storage systems for microorganisms that were thought to enter as vegetative and resting cells, transported by wind-blown particles, aerosols and ice crystals [8]. However, a number of studies have challenged this view by examining the unique biodiversity of microorganisms in cold environments [9]–[11], their impact on the dynamics, composition and abundance of nutrients [12], their role in shifting surface albedo of snow and ice [13], and their influence on Arctic hydrochemistry [14]. Microorganisms in the Arctic also mediate the transformation of contaminants, such as Hg [15]–[17]. In turn, environmental factors can also influence microbial community structure. In both field and laboratory studies, Hg was shown to alter community structure and function with the enrichment of resistant populations and changes in contaminant metabolism in sediments [18]. However, the microbial community dynamics in complex cold ecosystems remain to be fully explored. A recent report on a seasonal Arctic snowpack showed a rapidly changing chemical environment along a seasonal gradient and that Hg transformations were occurring within the snowpack [7]. If the snowpack chemistry is dynamic, then it is possible that the microbial communities inhabiting this system are as well. In order to gain insight into Arctic ecosystem functioning, the interaction among its different components that make up this system needs to be determined. While Arctic snowpack chemistry has been studied in detail [6], [7], knowledge about microbial community structure is lacking despite its involvement in ecosystem-level processes [19]. A recent report by Hell et al. (2013) highlighted the presence of a diverse and dynamic microbial community in melting glacial snowpacks and suggested post-depositional changes in community structure [20], while Larose et al. (2013) found that microbial communities inhabiting the snow cover demonstrated dynamic shifts in their functional potential to carry out several different pathways of the nitrogen cycle [21].

Here, we report the results of a two-month field study in a high Arctic site, Svalbard, Norway (79°N). Collected samples were analysed by determining the snowpack and meltwater chemistry and following shifts in microbial community structure with a 16S rRNA gene microarray. We investigated the relationships between the chemical and microbial data by co-inertia analysis. We also explored changes in the microbial community associated with seasonal atmospheric Hg deposits by using metagenomic approaches to quantify mercury resistance genes in samples.

## Methods

### Sampling

Samples were collected during a spring research campaign held between April 16^th^, 2008 and June 8^th^, 2008 at Ny-Ålesund in the Spitsbergen Island of Svalbard, Norway (78°56′N, 11°52′E). Access to the site was granted by the Svalbard Science Forum (project registration number 4762) chaired by the Research Council of Norway and field studies did not involve endangered or protected species. The field site, consisted of a 50 m^2^ perimeter with restricted access to reduce contamination from human sources located along the south coast of the Kongsfjorden, which is oriented SE-NW and open to the sea on the west side. The Kongsfjorden was free of sea ice throughout the campaign.The snowpack consisted of seasonal snow that accumulated above the soil. Twice a week, a shallow pit (45 cm at the beginning of the field season) was dug and both surface (3 cm) and basal samples (10 cm above the ground) were collected for cation/anion chemistry, mercury and microbial analyzes. A total of 39 samples were collected. For cation/anion chemistry measurements (major, minor ions and organic acids), samples were collected in sterile cuvettes and stored at −20°C until analyzed. For Hg analyzes, snow was collected in acid-washed 250-mL glass Schott bottles (based on the cleaning protocol described in [22]) and subsampled for bioavailable Hg (BioHg). For methyl mercury (MeHg) measurements, samples were collected in 125-mL acid-washed Teflon-coated low-density polyethylene bottles and stored frozen until analyzed. Meltwater was collected in acid-washed 250-mL glass Schott bottles from streams that formed starting June 1^st^, 2008. Snow and meltwater chemistry as well as sample handling and analyses have been described previously. BioHg was determined using a *mer-lux* bioreporter [23]. MeHg, total mercury (THg) and organic and inorganic ion data are presented in Larose et al. 2010b [7]. Samples for microbial analyzes were collected in three 3-L sterile sampling bags using a sterilized Teflon shovel for a total of 9 L of snow equivalent to about 3 L of water depending on snow density. To minimize contamination, Tyvex® body suits and latex gloves were worn during sampling and gloves were worn during all subsequent sample handling.

### Microbial Sample Processing

Samples were processed immediately in the field laboratory. Snow samples were left to melt for 6 hours at room temperature and the resultant water was filtered with sterile 0.22-µm pore size, 47-mm diameter filters (Millipore) using a sterile filtration unit (Nalge Nunc International Corporation). Meltwater samples were filtered immediately. Filters were stored in sterile bead-beating tubes at −20°C until analyzed. Procedural blanks were performed by filtering Nanopure water (Siemens) using the same procedure.

### DNA Extraction

Filters were cut up and placed in a Fastprep® bead-beating tube (Lysing matrix E, MP Biomedicals) to which 1 mL of DNA extraction buffer [24] and 20 mg.mL^−1^ lysing enzyme (*Trichoderma harzianum*, Sigma L1412) were added. Tubes were incubated at room temperature for 1 hour and then frozen at −20°C overnight. The frozen tubes were incubated at 65°C for 30 minutes and placed in a Fastprep® bead-beater (MP Biomedicals) at 5.5 m/s for 30 seconds. DNA was extracted from the aqueous phase with an equal volume of chloroform:isoamyl alcohol (24∶1) and precipitated with isopropanol.

### DNA Taxonomic Microarray Analysis

The Agilent Sureprint Technologies microarray format was used, and consisted in 8 identical blocks of 15,000 spots each on a standard glass slide format 1″×3″ (25 mm×75 mm). Each spot is formed by *in situ* synthesis of 20-mer oligoprobes that occur at least in triplicate within each block. Five slides were used for the hybridization of all samples (39 individual blocks, 1 block per sample). Probes were designed to target the *rrs* gene at different taxonomic levels (1469 genera, 286 families, 118 orders, 57 classes, 36 Phyla based on NCBI taxonomy) from the *Bacteria* and *Archaea* phylogenetic tree using the ARB software package (phylogenetic microarray target) (for probes see reference 27 and http://www.genomenviron.org/Research/Microarrays.html). We chose to design 20-mer long probes with a melting temperature range of 65±5°C and a weighted mismatch of less than 1.5. This array has been used to observe changes in microbial diversity in soils as a function of DNA extraction procedures [25] and has been compared to snow microorganism clone libraries [9].

The *rrs* genes were amplified by PCR from total DNA extracted from each sample, using primers pA (5′ AGAGTTTGATCCTGGCTCAG 3′) and pH-T7 (5′ AAGGAGGTGATCCAGCCGCA 3′) [26] and the Illustra Hot Start Mix RTG PCR kit (GE Healthcare). The 25-µL volume PCR reaction mix contained 0.6 µm of each primer, 2 µl DNA or 2 µl sterile water for the negative controls. The PCR conditions were 3 min at 94°C, followed by 35 cycles of 45 s of denaturation at 94°C, 45 s of annealing at 55°C, and 90 s of elongation at 72°C. After a final 5-min extension at 72°C, PCR products were separated by 1%-agarose gel electrophoresis, purified using the *NucleoSpin*® *Extract* II kit (Clontech) and transcribed. *In vitro* transcription was carried out at 37°C for 4 hours in 20 µL that contained 8 µL of the purified PCR product (50 ng.µL^−1^) and 12 µL of the following mix: T7 RNA buffer (5X), DDT (100 mM), the four NTPs (10 mM each), RNasin (40 U.µL^−1^), T7 RNA polymerase (1 µL) and Cy3-UTP (5 mM). During transcription, the fluorescent Cy3-UTP, which emits light at 532 nm, is incorporated during the PCR to label the RNA. RNA was purified using the Qiagen RNeasy mini Kit according to the manufacturer’s instructions and quantified with a nanophotometer. Samples were then subjected to chemical fragmentation by addition of 5.7 µL of a Tris Cl (1 mM) and ZnSO_4_ (100 mM). Samples were incubated for 30 minutes at 60°C. Fragmentation was stopped by placing the tubes on ice. EDTA (500 mM) was added to each tube (1.2 µL) followed by 1 µl RNAsin (40 U.µL^−1^) after a minute of incubation at 25°C. The RNA solution was then diluted to 5 ng.µL^−1^ and a hybridization mix was prepared (v/v ratio) in 50 µL with 2X GeX Hyb Buffer (Agilent). A total of 100 ng of RNA were then deposited on the slide and incubated at 60°C overnight in the Agilent Hybridization Oven. Microarrays were washed with the GE wash Buffer kit (Agilent) according to the manufacturer’s instruction and dried by one minute immersion in acetonitrile.

### Microarray Scanning and Data Processing

An Innoscan 700 scanner (Carbonne, France) was used for scanning microarray slides according to the manufacturer’s instructions. Raw hybridization fluorescence signals for each spot were determined based on the signal-to-noise ratio (SNR), which was calculated following log-transformation of data, using the following formula: SNR = (signal intensity − background)/standard deviation of background. Since at least three replicates are present for all oligonucleotide probes, outliers were eliminated when any individual spot was greater than two standard deviations from the average of all replicates. ANOVA was used to evaluate positive probes from the results for all microarray data from one experiment. Since probes have different phylogenetic depth, genera described here were those for which all relevant probes were positive. While all of the probes could not be independently verified, many of them were validated by the application of DNA from pure cultures [27].

### q-PCR Screening for *merA* Genes

The primers were designed based on the alignments of known *merA* gene sequences. A total of 105 *merA* genes were retrieved from the NCBI reference sequence database [28] (http://www.ncbi.nih.gov/blast) and aligned in Clustal W [29]. Five non-degenerate primers were designed to cover most of the known *merA* diversity, using Primer Select (DNASTAR, Inc., Madison, WI). Positive controls were constructed for primer sets by a gene shuffling technique outlined in [30]. Briefly, an initial PCR cycle was carried out with two long oligonucleotide primers but without the addition of any other DNA template. Hybridization of the primer complementary regions led the polymerase to synthesize double strands of DNA complementary to the whole targeted DNA region. The second step involved the amplification of the synthetic sequence using the first reaction products as DNA template in a conventional PCR reaction. The initial cycle was carried out in the presence of 50 ng of each of the two long oligonucleotide primers, 1X Titanium Taq PCR Buffer, 0.2 mM deoxynucleoside triphosphates, 1 µL Titanium Taq DNA polymerase (Clontech-Takara Bio Europe, Saint-Germain-en-Laye, France) and 1 µL of T4 gene 32 protein (Roche Diagnostics S.A.S., Meylan, France). The PCR amplification was performed as a single-tube reaction in a Thermal Cycler (Biometra T1 Labgene scientific instrument, Archamps, France) as follows: 6 min at 96°C, followed by 35 cycles of 95°C for 1 min; 53°C for 30 s; and 68°C for 40 s, with a final extension at 68°C for 6 min. The second step was performed using the same reaction mix as for the initial cycle, except that 50 ng of the first reaction product as DNA template and 0.5 µM of forward and reverse primers were used [30]. Of the 5 primer sets tested, only one was able to amplify *merA* from our Arctic samples. The primer sets for *merA* amplification and gene shuffling are listed in Table 1.

**Table 1:** Short and long primers used for gene shuffling. The positive control used for q-PCR is also listed.

Primer type		5′ to 3′
Short primers	MerAF	GAAGCGGGTGAACTGATCC
	MerAR	TCGTCAGGTAGGGGAACAAC
Long primers	F	GAAGCGGGTGAACTGATCCAGACGGCGGCTCTGGCCATTCGCAACCGCATGACGGTGCAGGAACTGGCCGAC
	R	ATCGTCAGGTAGGGGAACAACTGGTCGGCCAGTTCCTGCACCGTCATGCGGTTGCGAATGGCCAGAGCCGCCG
Positive control	MerA	GAAGCGGGTGAACTGATCCAGACGGCGGCTCTGGCCATTCGCAACCGCATGACGGTGCAG

q-PCR analyzes were carried out in triplicate in 20 µL reaction volumes containing 10 µL of Quantace SensiMix™ Plus SYBR® (Quantace), 0.5 µL of each primer (10 µM) and 2 µL of DNA (concentrations normalized to 3 ng.µL^−1^), on a Rotor-Gene 3800 (Corbett Research, Sydney, Australia). The amplification protocol consisted of an initial denaturation phase (95°C for 10 min), followed by 45 cycles of denaturation (95°C for 15 s), annealing (57°C for 20 s) and elongation (72°C for 20 s). The integrity of q-PCR products was confirmed by melting curve analyses, from 50°C to 99°C. Standard curves were calculated based on gene copies per µL using 10-fold increments and were adjusted from 10^8^ to 10^1^ gene copies.µL^−1^. Amplicons were sequenced in order to verify the specificity of the primers.

### Statistical Analysis

Principal component analysis (PCA) was carried out on chemical data following log-transformation and correspondence analysis (COA) was used to analyze microbial data, since probe intensities were not normally or log-normally distributed. Results are plotted in two dimensional space based on the scores of the first two principal components. Co-inertia analysis (CIA) [31] was used to study the relationships between chemistry and microbial community structure. While different methods exist for studying complex data sets, CIA was specifically developed to study species-environment relationships and can be used to study spatial and temporal variations simultaneously [32]. The snowpack is not a completely closed system, as it experiences changes in structure and physics. Therefore, time-series analysis, based on the assumption that successive values in the dataset represent consecutive measurements taken at equally spaced time intervals, is not appropriate for describing interactions within the snow.

The objective of CIA is to create a factorial plane deforming as little as possible the structure of each dataset and enabling a simultaneous ordination of the data. Coupling the correlation of snow/meltwater chemical characteristics and community structure data matrices produced the co-inertia axes by projecting variables and sampling plots in a new factorial map. CIA is described in detail elsewhere [31], [33]. A randomization test of 10,000 permutations was carried out to verify the significance of the co-structure (Monte Carlo test). PCA, Monte Carlo and co-inertia tests were performed in R (The R Project for Statistical Computing http://www.r-project.org) using the ade4 software package [34]. Following CIA, samples were grouped using K-means, a method of cluster analysis that aims to partition observations into clusters such that the sum of squares from points to the assigned cluster centers is minimized [35]. A randomization test of 10,000 permutations was performed. Prior to clustering, samples were centered and data was normalized as a function of the contribution of each axis in explaining overall variance of the CIA. K-means is an unsupervised clustering method implying that the number of groups (the K number) has to be set. To choose a relevant K, we relied on the “gap” statistics (defined as the difference of the sum of the pairwise distances in each group and its expectation) [36]. The “gap” statistic was computed with a K value picked over a range from 1 to 12 following 1000 iterations (using the lga R package [Justin Harrington, 2012, lga: Tools for linear grouping analysis (LGA). R package version 1.1–1 http://CRAN.R-project.org/package=lga] implementation) reached a maximum for K = 8. Linear regression analysis between chemical species and gene copy numbers was carried out using JMP 9.0 software (SAS Institute, 2003). Metabolic potential and phyla/class distributions were carried out in MEGAN version 4.69 [37].

## Results

This study acquired a large pool of physical chemical and microbiological data associated with each sample and there were several samples from different snow types at different times. Sample description and some of the raw chemical analyses (a full description is provided in [7]) and the taxonomic data are provided in the supplementary information section (see Tables S1–S3 in File S1). This data was organized by the application of a co-inertia analysis.

### Co-inertia Analysis between Snow/Meltwater Chemistry and Microbial Community Structure

**Figure 1:**
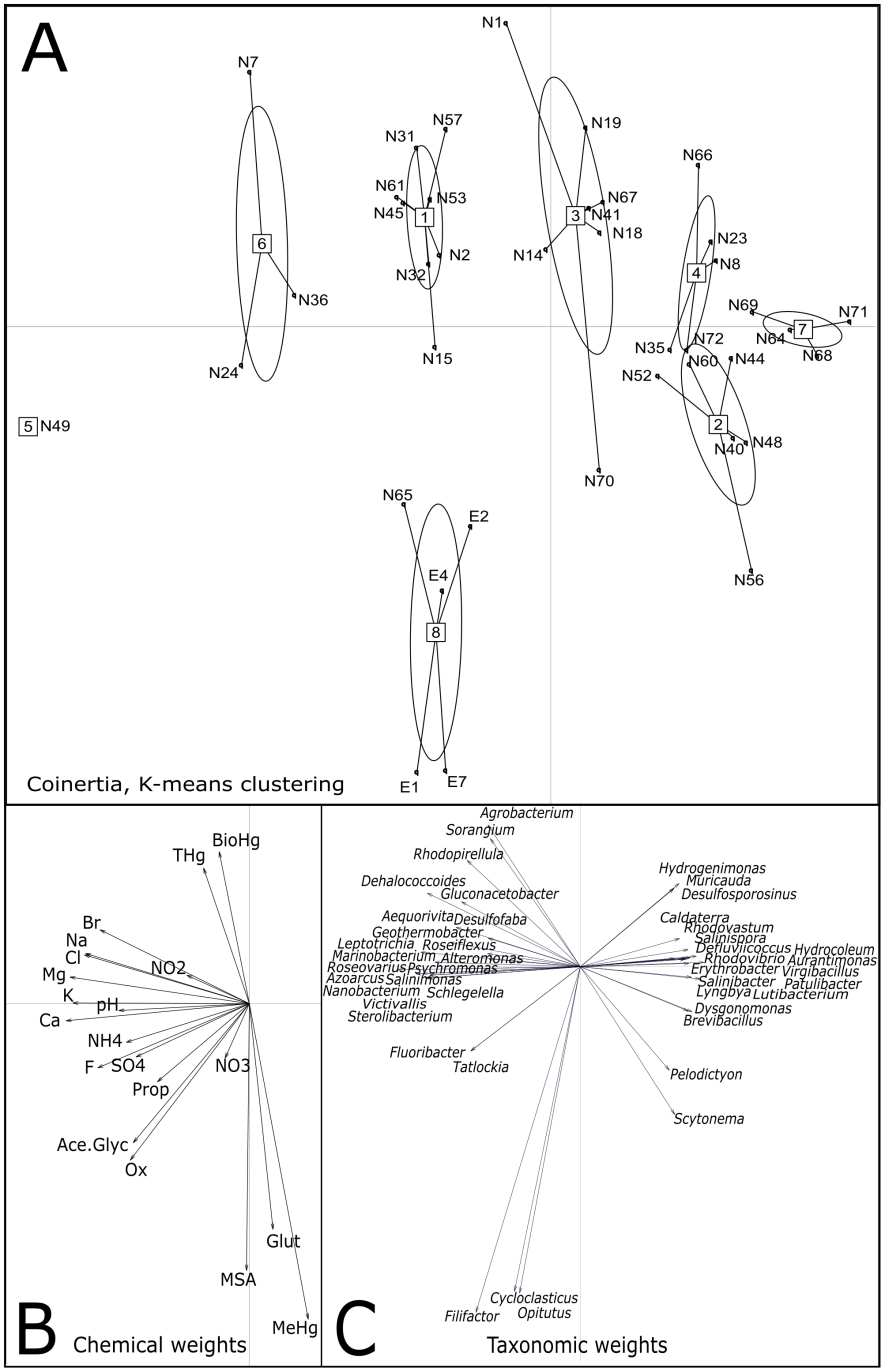
Co-inertia analysis of the chemical and microbial data. (1A) K-means clustering output. Each of the eight groups is numbered inside square boxes and the 39 samples are indicated and linked to the boxes. The ellipses represent group clusters based on K-means clustering. (1B) Main chemical vectors that affect sample ordination. The lengths of the vector arrows represent the influence of the given parameter on the co-structure of the CIA. Anions and cations are represented by their chemical symbols and organic acids are given as: Prop (propionate), Ox (oxalic acid), Ace.Glyc (acetate-glycolate), MSA (methylsulfonic acid) and Glut (glutaric acid). (1C) Probes showing the greatest influence on the ordination. The lengths of the vector arrows represent the influence of the given parameter on the co-structure of the CIA.

A co-inertia analysis (CIA) was carried out on snow/meltwater chemistry and microbial community structure (Figure 1). The permutation test revealed a significant relationship between the snow/meltwater chemistry and the microbial community structure (*p* = 0.0058, RV = 0.324). The RV-coefficient represents the correlation between both datasets and varies between 0 and 1; the closer the coefficient is to 1, the stronger the correlation between the datasets. The first four eigenvalues of the co-inertia analysis accounted for 51.4, 15.8, 9.7 and 9.1% of the explained variance, respectively, and all were used to estimate cluster inference, although only the first two are presented in the figures. The chemical and microbial datasets explained 53.5 and 46.5% of the total variance, respectively.

In the graphical representation presented in Figure 1A, we grouped the 39 samples based on the K-means analysis (10,000 permutations) which reflects their ordination in both the chemical and microbial datasets. Variables are represented on correlation circles and correlations between the original data sets and the score or latent variable vectors are computed so that highly correlated variables cluster together in the resulting figures. Therefore, interactions between two types of variables can be identified in addition to identifying the relationship between variable clusters and associated sample clusters. The chemical parameters that had the most influence on the co-structure as observed by the lengths of the vector arrows in Figure 1B formed four major axes: 1) BioHg (and total Hg); 2) MeHg (and marine biogenic molecules glutarate and MSA); 3) pH and organic acids; and 4) ions. The bacterial genera (based on the phylogenetic microarray) with the most influence on the co-structure number around 50 (Table 2) and are presented in figure 1C in order to visualize their relative importance (length and direction of vectors) in the different groups shown in figure 1A.

**Table 2:** Groups of samples and their characteristics as determined by co-inertia analysis.

Group	Sample type	Chemical drivers*	Most influential probes
**1**	Basal snow	High nitrate, high ion,salinity 0.7‰	*Actinobaculum, Micrococcus, Cyanothece*, *Halothece, Halobacillus, Neisserria,Glucunobacter*
**2**	Warm, wet surface snow	High MeHg, Glut, MSA,low ions salinity 0.05‰	*Oscillatoria, Planktothrix, Pelodictyon Phormidium, Microcoleus, Microcystis, Synechococcus, Synechocystis, Scytonema*
**3**	Fresh surface snow,some basal samples	High Hg and BioHg,low pH, salinity 0.3‰	*Agrobacterium, Sorangium, Sulfobacillus, Thiobaca, Thiococcus*
**4**	Mainly surface snow	Low ion (salinity 0.06‰)	*Desulfosporosinus, Hydrogenimonas, Muricauda*
**5**	Basal sample,isothermal snowpack	High ion (salinity 23‰)	*Achromatium, Acidimicrobium, Aeromonas, Agarivorans, Roseovarius*
**6**	Fresh surface snow (depositionevent), basal snow samples	High ion (salinity 8‰)	*Psychromonas, Alteromonas, Azoarcus, Exiguobacterium, Leptotrichia, Marinobacter, Pelobacter, Roseiflexus, Ruminococcus*
**7**	Late season, drysurface snow	Low ion (salinity 0.02‰)	*Flexibacter, Arenibacter, Lentisphaera,Hydrocoleum, Lyngbya, Virgibacillus, Oscillochloris, Rhodovibrio, Desulfobacca*
**8**	Meltwater samples	High organics, elevatedpH, low Hg	*Flavobacter, Filifactor, Cycloclasticus, Opitutus, Xanthobacillum, Alkalibacterium*

* MeHg, Glut, MSA, Hg, BioHg represent methylmercury, glutarate, methylsulfonic acid, mercury and bioavailable mercury, respectively.

**Figure 2:**
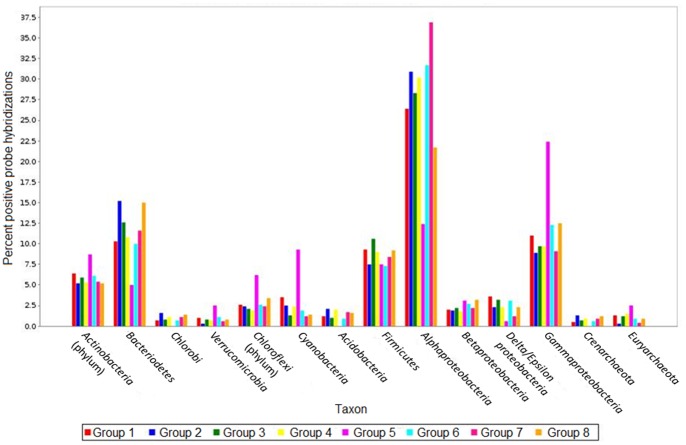
Distribution of major phyla/classes among the eight groups of samples.

**Figure 3:**
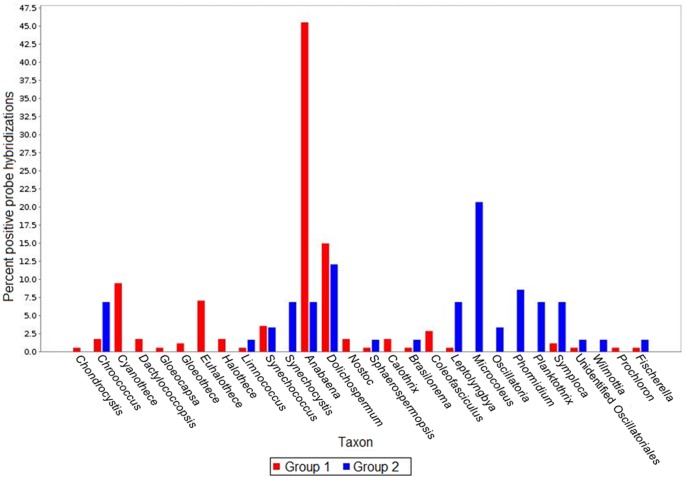
Distribution of cyanobacterial taxa between Groups 1 and 2.

The “gap” statistic computed with a K value picked over a range from 1 to 12 following 1000 iterations reached a maximum for K = 8. Then, the K-means analysis (Figure 1A) was performed in order to infer the 8 most consistent groups of samples based on both their chemical and taxonomic characteristics (Table 2). For each group, we estimated the coverage of the potential diversity of the different phyla identified by the microarray (see Table S3 in File S1). The coverage was calculated as the percentage of the average number of positive probes per snow sample group (probes that fluoresced upon analysis) to the total number of probes targeting all bacterial phyla and classes. An overview of phyla/class distribution among samples is presented in Figure 2. Most positive probes were associated with *Alphaproteobacteria*, followed by *Bacteroidetes* and *Gammaproteobacteria*. Coverage was highest in the surface snow samples (Groups 3 and 6), which contained almost all of the genera detected in all of the other samples. Basal snow from the isothermal snowpack (Group 5) had the lowest coverage with no positive hybridizations for many of the most abundant probes at the genera level, yet, Group 5 had the highest percentage of probes associated with *Gammaproteobacteria*, *Cyanobacteria*, *Chloroflexi*, *Actinobacteria* and *Verrucomicrobia* of all the groups (see Table S2d in File S1). Probes associated with *Cyanobacteria* and *Actinobacteria* were also relatively elevated in the other basal snow group (Group 1). This group also had the highest percentage of *Delta/Epsilonproteobacteria*. The oligonucleotide probes (those with positive hybridization of nucleotides representing different taxonomic levels on the microarray) that had the most effect on sample ordination as determined by the CIA were identified (Table 2). Probes targeting certain genera such as *Brevundimonas*, *Sulfitobacter*, *Rhizobium*, *Mesorhizobium* (*Alphaproteobacteria*), and *Pseudomonas* (*Gammaproteobacteria*) were the most abundant and detected in almost all samples (see Tables S2a–e in File S1). An example of differences between samples is provided for the basal snow (Group 1) and the wet surface snow samples (Group 2) relative to the distribution of cyanobacterial taxa (Figure 3).

**Figure 4:**
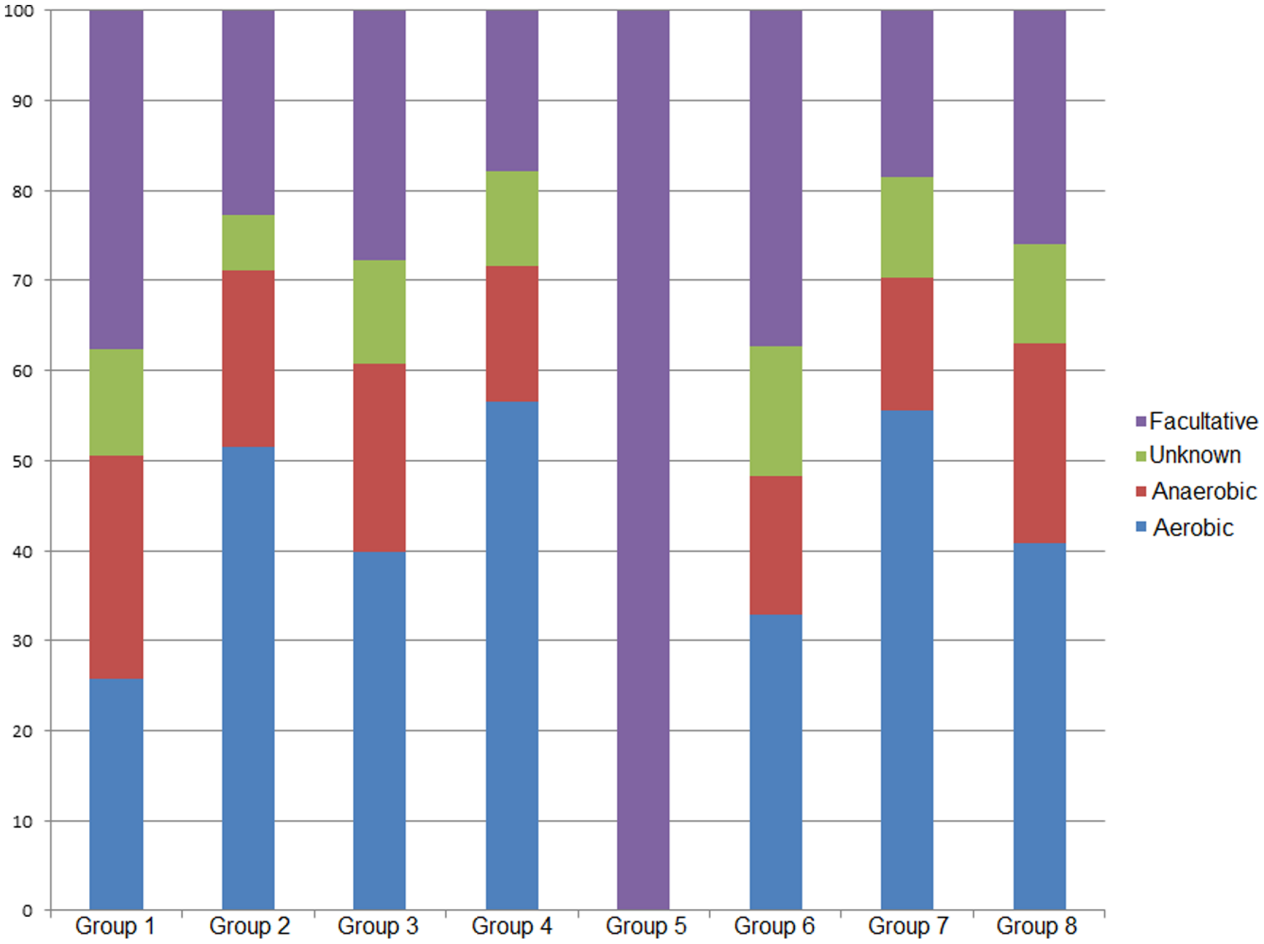
Metabolic potential of the snowpack. The proportion of each type of analyzed metabolism (aerobic, anaerobic, facultative and unknown) is given for each of the eight groups.

Metabolic information (aerobic/anaerobic/facultative/unknown) was assigned to all positive probes in MEGAN and compared among groups (Figure 4). Basal snow samples (Group 1) had the highest percentage of probes associated with bacteria known for their anaerobic metabolism and the second highest for those associated with facultative anaerobic metabolism. All surface samples (Groups 2, 4 and 7) had the highest amount of probes associated with aerobic metabolism. All the reads in the basal snow from the isothermal snowpack (Group 5) were associated with bacteria capable of facultative anaerobic metabolism.

### Presence of *merA* Genes

**Figure 5:**
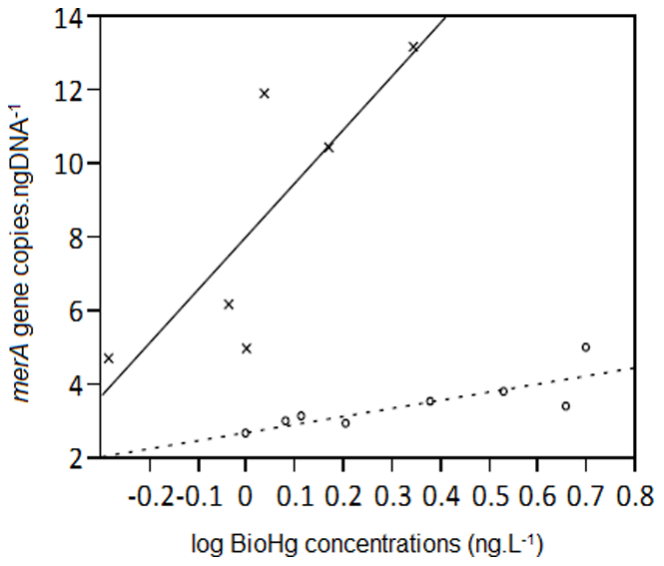
Linear correlation between *merA* gene copy number (copies.ngDNA^−1^) and BioHg concentrations (log ng.L^−1^). Crosses represent samples from Group 2, and circles samples from Groups 3 and 7.

Based on the observation that mercury has an influence on community structure, we performed q-PCR analysis on all samples in order to determine whether *merA* genes were present. We could only detect *merA* genes in samples taken after springtime atmospheric mercury depletion events (AMDEs) and mainly in surface snow samples. The first AMDEs occurred in March (before sampling commenced) and the others were recorded between the 17^th^ and 25^th^ of April [38]. A significant linear correlation was detected between gene copy number and bioavailable mercury (BioHg) concentrations in surface snow samples with distinctive patterns as a function of sample group (Figure 5). The warm wet surface snow exhibited a steeper increase in *merA* genes with BioHg than did the surface snow during drier seasons and colder temperatures.

## Discussion

### Microbial Community Structure and Metabolic Potential

Based on the 16S rRNA gene microarray results, we were able to describe the snowpack microbial community and its dynamics. We focused on changes in total community structure and metabolic potential. Probes detected Gram-negative aerobes that have been detected in diverse cryosphere environments [11], [39]–[41]. Related phylotypes have also been reported in geographically-diverse cold environments [42], suggesting that adaptation for survival, persistence and activity at low temperatures might be common traits of these species with potentially similar underlying adaptive strategies [11]. The bacterial genera most frequently reported in cryosphere environments are *Proteobacteria* (*Alphaproteobacteria*, *Betaproteobacteria* and *Gammaproteobacteria*), *Bacteroidetes*, low and high G+C Gram-positive genera, and *Cyanobacteria* [11], [39]–[41], [43]. Although mostly aerobic bacterial genera were detected in the snow (Figure 4), the presence of anaerobes may be explained by anaerobic microniches within the snow. Anaerobic activity was not tested directly. The highest proportion of reads associated with anaerobic metabolism in the snowpack was observed in basal samples (Group 1). The basal samples were chemically enriched by early season surface snow due to meltwater percolation to the base of the snowpack, which may have result in a reduction in the oxygen content. (Melt started around the 20^th^ of May, see [7] for more details).

### Colonization-where are they from?

One important pathway for colonization of Arctic snowpacks may be precipitation. Biological particles, such as proteins or proteinaceous compounds, play a significant role in the initiation of ice formation, especially when cloud temperatures are warm [44]. Fresh snow samples (Groups 3 and 6) had the highest microbial richness suggesting a higher diversity compared to aged snow. Many of the positive probes that were predominant in fresh snow samples or samples collected during deposition events targeted genera that are known to be plant pathogens, such as *Agrobacterium* [45]. Some plant pathogens are known for their ice nucleating activity, which may explain their increased detection in freshly fallen snow [46]. We also found ice nucleation genes and proteins in *Agrobacterium* in genome datasets. However, these genera were no longer detected after deposition, which may suggest environmental, post-depositional selection, a phenomenon also reported by Hell et al. [20]. However, precipitation is not the only possible colonization pathway as others, including aerosols from the fjord, dust particles from the surrounding mountains, human and animal sources, and soil might also provide microbial inputs. However, while the chemistry of the snowpack contained a marine signature, the microbial community generally did not (except for Groups 5 and 6).

### Linking Chemistry and Microbial Community Structure: Post-depositional Selection?

The dynamic nature of the snowpack in terms of both chemistry and community structure is revealed by the clusters that evolved along a seasonal gradient and the surface and basal samples that evolved differently over time. This analysis demonstrated a significant co-structure based on a Monte Carlo test with 10,000 permutations (*p = *0.0058) linking the snow pack chemistry and microbial community structure. Each snow sample group had its own taxonomic signature that was linked to changes in environmental conditions, such as specific chemical parameters. However, the direction of the cause-effect is not readily apparent.

### Marine Salts, Organic Acids and pH

The co-inertia analysis determined the major chemical and microbial vectors that influenced the possible interactions. Marine salts (inorganic ion axis) drove the ordination of samples and the relative abundance of probes targeting specific phylotypes increased as a function of ion concentration (Figure 1A–C). Two clusters were characterized by significantly higher salt concentrations, with salinity values of brackish water for basal snow (Group 5 at 23 ‰ salinity) and fresh surface snow during a deposition event (Group 6 at 8 ‰ salinity). The ordination of both groups was influenced by probes that target genera whose members are cold-adapted and commonly observed in sea ice such as *Marinobacter*, *Psychrobacter* [47], *Roseovarius* [48] and *Achromatium* [49] (Table 2), although the community structure and metabolic potential in both groups differed. The community structure of highly saline basal snow (Group 5) at the class/phylum level closely resembled that of sea ice, with *Gamma-Proteobacteria* dominating, while that of fresh surface snow during a depositional event (Group 6) was similar to the other snow samples (Figure 2).

Organic acids and pH represented another important vector and were linked to meltwater samples (found in Group 8). Short-chained acid (with one or two carbons) concentrations increased as the season progressed and the snowpack began to melt. pH has been reported as one of the main factors determining community structure in soils [50], but, to the best of our knowledge, this is the first report of pH as a potential driver of community structure in snow samples. The probes that drove the ordination of this group target psychrotolerant, halotolerant and obligately alkaliphilic genera such as *Alkalibacterium* [51] and *Opitutus* [52].

### Nitrogen and Sulfur Cycling in the Snowpack

As temperatures increased, the snowpack became both warmer and wetter. In the warm, wet snow from both the surface and the base of the snowpack (Groups 1 and 2), many of the probes that had the most impact on sample ordination targeted genera belonging to the *Cyanobacteria* phylum (Table 2). In addition, both groups had among the highest percentage of positive probe hybridizations associated with *Cyanobacteria* (Figure 2), with the exception of Group 5. However, we observed differences in *Cyanobacteria* taxa between these two groups with a predominance of non-heterocystous *Oscillatoriales* in surface samples (Group 2; Figure 3) and heterocystous *Nostocales* in basal samples (Group 1; Figure 3). Probes targeting halophilic genera that have been reported to metabolize nitrogen, such as *Anabaena* [53], *Cyanothece* and *Halothece*, were predominant in the basal snow group (Group 1), whose ordination was driven by NO_3_^−^ and NH_4_^+^ concentrations (Figure 2). Nitrogen availability is an important factor of ecosystem productivity [54] and biological nitrogen fixation is a recognized function carried out by *Cyanobacteria* in diverse environments including terrestrial and aquatic Antarctic and Arctic habitats [55], [56]. Based on nitrogen isotope fractionation data, microorganisms were might not only be involved in nitrogen cycling in Arctic snowpacks during winter, but also might be the main drivers of this process [57].

The surface snow samples (Group 2) were relatively low in NH_4_, NO_3_, chloride and other inorganic ions and their average salinity (0.05‰) was similar to values observed for freshwater (<0.5‰). The probes with the most influence on sample ordination and specific to this group targeted sulfur-metabolizing photoautotrophs, such as *Microcystis* [58] and *Synechococcus* [59]. All living organisms require sulfur, which can be assimilated for the synthesis of proteins and essential cofactors from either inorganic sources like sulfate and thiosulfate or organic sources like sulfate esters, sulfamates and sulfonates [60]. The ordination of Group 2 was driven by high levels of methylsulfonic acid (MSA), a derivative of dimethylsulfoniopropionate (DMSP), which is a major source of sulfur for bacteria in the marine environment [61]. DSMP is an organic sulfur compound produced by algae as an osmoprotectant, predator deterrent and antioxidant [62] that is released upon cell senescence, viral attack or in response to stress [63]. Used by bacteria as a carbon and sulfur source, it could also serve as an osmo and cryoprotectant [64]. DSMP can undergo either direct uptake by bacteria via specific transporters [63] or degradation by extra-cellular enzymes. The high concentrations observed in our samples suggest a close source for DMSP [7] that might have been deposited after algal blooms occurred in the fjord near our sampling site or produced by organisms in the snowpack, since DMSP production has also been found to be predominant in some freshwater environments [65], [66]. Although generally attributed to algal production, DMSP production in cyanobacteria, such as *Microcoleus* [67] and *Synechocystis* [68], has also been reported. Their detection in surface snow samples (Group 2) might support *in-situ* DMSP production.

The group of surface snow samples collected in June (Group 7) had the lowest salinity of all the groups and were collected after the snowmelt started. We observed a shift in community structure from Group 2 to Group 7, with less *Cyanobacteria* detected in June. This may be linked to snowpack water content and loss of certain taxa due to meltwater elution. The probes that had the most influence on the sample ordination of Group 7 targeted psychrophilic and halophilic organisms such as *Arenibacter* [69] and *Lentisphaera* [70], which might constitute a sea spray signal in the surface snow. Probes that targeted sulfur-metabolizing genera also had an impact on the sample ordination of Group 7, although they differed from those found in Group 2 samples as they targeted non-photoautotrophic genera such as *Hirschia* [71] and *Marichromatium* [72] in addition to aerobic bacteriochlorophyll-containing bacteria such as *Erythrobacter* [73].

### Contaminant Cycling

Based on the CIA, MeHg and BioHg constituted important vectors in determining sample ordination. At the beginning of the sampling period, we recorded several atmospheric mercury depletion events (AMDEs) that resulted in a 30-fold increase of THg (the sum of all Hg species) and a 6-fold increase of BioHg concentrations in snow surface samples (maximum values were 150 ng.L^−1^ THg and 20 ng.L^−1^ BioHg) [7]. In a laboratory study with soil microcosms, an immediate decrease in microbial diversity after the addition of 25 µg Hg^2+^.g^−1^ to the soil was observed [74]. This decrease in diversity was due to a shift in community structure by the enrichment of Hg-resistant members, but was subsequently recovered. Hg at concentrations (8 mg of Hg.L^−1^) far above the natural ng.L^−1^ range was also able to induce changes in community functional and genetic structures based on protein-fingerprinting and automated ribosomal intergenic spacer *analysis* (ARISA) [75]. While Hg appears to influence community structure, this relationship is likely bidirectional. Based on a significant negative correlation between BioHg and MeHg, biotic methylation of Hg is probably occurring in the Arctic [7]. Therefore, microorganisms can potentially alter their chemical environment through the metabolism and transformation of elements or contaminants. In order to cope with the toxicity of Hg^2+^ and MeHg, bacteria have developed specific resistance mechanisms. For example, bacteria possessing the *mer* operon are able to detoxify Hg^2+^ via a MerA reductase that transforms mercuric ions into elemental volatile Hg (Hg(0) [17]. Other bacteria are able to transform organic mercury compounds such as MeHg using a second enzyme, MerB, that cleaves the mercury- carbon bound. In addition, *merA* genes have been detected in diverse environments including soil [76], Siberian permafrost [77] and Arctic biofilms [15] as well as in specific bacteria [17] and archaea [78]. We only detected *merA* genes in samples collected after the AMDE period. This observation might be due to relative increase of mercury resistant bacteria in the snow microbial community during Arctic springtime mercury deposition events [79]. Based on our q-PCR results, *merA* gene copy number was positively correlated to BioHg concentrations in the snowpack and the response appears to be group specific. These group differences might be related to changes in community structure or to the rapid growth of Hg-resistant microorganisms. Competing processes leading to modifications in the Hg levels could potentially be performed by different members of the same community in the snowpack: some microorganisms might be methylating Hg to MeHg, while others might be reducing BioHg via *merA*.

## Conclusions

We have shown that snowpack/meltwater chemistry and microbial community structure are linked and that the snowpack is a dynamic habitat that undergoes changes during the spring. In addition, mercury in both bioavailable and methylated forms influenced microbial community structure, even at low doses. Finally, linking a 16S rRNA gene microarray approach to chemical data provided insights on interactions in complex ecosystems between bacteria and environmental chemistry without the need for bacterial cultivation. While our results are from one field season and may not be extrapolated to other environments, they provide a basis for further studies on the interaction between chemistry and microbial community structure.

## Supporting Information

File S1 **This file contains Table S1–S3.** Table S1, Sample description with sampling dates, snow type and some chemical parameters. Table S2a, Fluorescence data (group 1) for the most abundant probes at the genera level. Table S2b, Fluorescence data (group 2) for the most abundant probes at the genera level. Table S2c, Fluorescence data (group 3) for the most abundant probes at the genera level. Table S2d, Fluorescence data (groups 4, 5, 6) for the most abundant probes at the genera level. Table S2e, Fluorescence data (groups 7, 8) for the most abundant probes at the genera level. Table S3, Positive hybridizations for different bacterial classes as a function of phyla/class. Values are given as averaged counts and percent of total per group.
(DOCX)
