# Supplementary material for: Interactions between Snow Chemistry, Mercury Inputs and Microbial Population Dynamics in an Arctic Snowpack
Source: PLoS One. 2013 Nov 25;8(11):e79972. doi: 10.1371/journal.pone.0079972 (PMC3839931; doi:10.1371/journal.pone.0079972)
Supplement: File S1 — This file contains Table S1–S3. Table S1, Sample description with sampling dates, snow type and some chemical parameters. Table S2a, Fluorescence data (group 1) for the most abundant probes at the genera level. Table S2b, Fluorescence data (group 2) for the most abundant probes at the genera level. Table S2c, Fluorescence data (group 3) for the most abundant probes at the genera level. Table S2d, Fluorescence data (groups 4, 5, 6) for the most abundant probes at the genera level. Table S2e, Fluorescence data (groups 7, 8) for the most abundant probes at the genera level. Table S3, Positive hybridizations for different bacterial classes as a function of phyla/class. Values are given as averaged counts and percent of total per group. (DOCX) [file pone.0079972.s001.docx]

Supporting Information

S1 Table 1: Sample description with sampling dates, snow type and some chemical parameters

| Sample Id | group | date | snow characteristics | pH | Hg ng/L | BioHg ng/L | MSA µmol/L | Cl µmol/L |
| --- | --- | --- | --- | --- | --- | --- | --- | --- |
| SVN31 | 1 | 06/05/2008 | *fresh surface snow* | 5.6 | 14.1 | 3.0 | 0.25 | 891.8 |
| SVN2 | 1 | 16/04/2008 | *basal snow* | 6.5 | 3.6 | 3.0 | 0.06 | 1197.8 |
| SVN15 | 1 | 23/04/2008 | *basal snow* | 5.6 | 1.4 | 1.2 | < DL | 2435.5 |
| SVN32 | 1 | 06/05/2008 | *basal snow* | 5.8 | 8.6 | 1.9 | < DL | 1243.9 |
| SVN53 | 1 | 23/05/2008 | *basal snow* | 5.5 | 3.5 | 0.9 | 0.05 | 1199.3 |
| SVN57 | 1 | 27/05/2008 | *basal snow* | 5.1 | 2.2 | 2.2 | < DL | 414.2 |
| SVN61 | 1 | 30/05/2008 | *basal snow* | 4.8 | 2.7 | < DL | < DL | 705.1 |
| SVN45 | 1 | 16/05/2008 | *basal snow* | 3.5 | 2.8 | < DL | < DL | 985.5 |
| SVN40 | 2 | 13/05/2008 | *warm, wet surface snow* | 5.1 | 3.7 | 1.5 | 1.38 | 30.7 |
| SVN44 | 2 | 16/05/2008 | *warm, wet surface snow* | NA | 3.1 | 0.9 | 1.30 | 30.9 |
| SVN48 | 2 | 20/05/2008 | *warm, wet surface snow* | 4.0 | 7.7 | 1.2 | 1.11 | 37.4 |
| SVN52 | 2 | 23/05/2008 | *warm, wet surface snow* | 4.3 | 4.6 | 0.5 | 3.07 | 207.0 |
| SVN56 | 2 | 27/05/2008 | *warm, wet surface snow* | 4.2 | 0.8 | 0.8 | 1.60 | 51.4 |
| SVN60 | 2 | 30/05/2008 | *warm, wet surface snow* | 4.7 | 0.9 | < DL | 1.75 | 67.5 |
| SVN1 | 3 | 16/04/2008 | *fresh surface snow* | 4.9 | 12.8 | 3.5 | 0.16 | 437.5 |
| SVN14 | 3 | 23/04/2008 | *fresh surface snow* | 6.1 | 85.4 | 3.4 | 0.08 | 1368.8 |
| SVN18 | 3 | 25/04/2008 | *fresh surface snow* | 5.5 | 58.5 | 7.3 | 0.05 | 623.3 |
| SVN19 | 3 | 25/04/2008 | *basal snow* | 4.5 | 3.7 | 2.4 | < DL | 331.5 |
| SVN67 | 3 | 04/06/2008 | *fresh surface snow* | 5.6 | 5.9 | 4.6 | 0.08 | 113.1 |
| SVN70 | 3 | 06/06/2008 | *basal snow* | 6.6 | < DL | < DL | < DL | 197.6 |
| SVN41 | 3 | 13/05/2008 | *basal snow* | 5.5 | 5.0 | 1.5 | < DL | 440.7 |
| SVN66 | 4 | 03/06/2008 | *surface snow* | 5.5 | 8.4 | 5.0 | 0.40 | 48.7 |
| SVN23 | 4 | 29/04/2008 | *surface snow* | 5.2 | 23.7 | 2.2 | 0.07 | 79.7 |
| SVN35 | 4 | 09/05/2008 | *surface snow* | 4.7 | 1.2 | 1.2 | 1.01 | 107.8 |
| SVN8 | 4 | 19/04/2008 | *basal snow* | 4.9 | 1.9 | 1.6 | < DL | 156.1 |
| SVN72 | 4 | 08/06/2008 | *surface snow* | 4.6 | 14.9 | 1.8 | < DL | 61.2 |
| SVN49 | 5 | 20/05/2008 | *basal snow, isothermal snowpack* | 6.1 | 10.9 | < DL | < DL | 35482.9 |
| SVN7 | 6 | 19/04/2008 | *fresh surface snow, deposition event* | 6.4 | 40.7 | 6.7 | < DL | 21456.5 |
| SVN24 | 6 | 29/04/2008 | *basal snow* | 5.1 | 5.2 | 1.2 | < DL | 8587.4 |
| SVN36 | 6 | 09/05/2008 | *basal snow* | 6.1 | 4.0 | 2.0 | < DL | 5694.3 |
| SVN64 | 7 | 02/06/2008 | *late spring dry surface snow* | 5.1 | 1.1 | < DL | < DL | 54.9 |
| SVN68 | 7 | 05/06/2008 | *late spring dry surface snow* | 5.5 | 4.7 | 1.3 | 0.09 | 17.3 |
| SVN69 | 7 | 06/06/2008 | *late spring dry surface snow* | 5.9 | 15.0 | 1.9 | < DL | 35.2 |
| SVN71 | 7 | 07/06/2008 | *late spring dry surface snow* | 5.4 | 5.5 | 1.7 | < DL | 31.1 |
| SVN65 | 8 | 02/06/2008 | *melting basal snow* | 6.4 | 3.3 | < DL | 0.57 | 1456.7 |
| SVE1 | 8 | 01/06/2008 | *meltwater* | 6.8 | 3.0 | 1.5 | 9.60 | 1265.1 |
| SVE2 | 8 | 01/06/2008 | *meltwater* | 6.9 | 3.2 | 1.8 | < DL | 320.6 |
| SVE4 | 8 | 04/06/2008 | *meltwater* | 6.9 | 3.1 | 0.9 | 0.59 | 872.8 |
| SVE7 | 8 | 07/06/2008 | *meltwater* | 6.5 | 5.6 | 1.2 | 2.02 | 291.0 |

S1 Table 2a: Fluorescence data (group 1) for the most abundant probes at the genera level

| Genera | SVN15 | SVN31 | SVN32 | SVN45 | SVN53 | SVN57 | SVN2 | SVN61 |
| --- | --- | --- | --- | --- | --- | --- | --- | --- |
| *Brevundimonas* | 25.3 | 75.6 | 53.1 | 0.2 | 2.0 | 0.0 | 29.9 | 0.0 |
| *Moorella* | 16.7 | 17.9 | 17.4 | 0.0 | 0.0 | 0.0 | 18.1 | 0.0 |
| *Pseudomonas* | 8.1 | 52.6 | 10.4 | 4.9 | 6.7 | 4.2 | 43.9 | 3.1 |
| *Mesorhizobium* | 9.7 | 15.6 | 24.8 | 4.6 | 1.3 | 0.0 | 12.8 | 0.9 |
| *Sulfitobacter* | 12.6 | 12.5 | 12.4 | 12.2 | 12.8 | 12.1 | 13.6 | 11.8 |
| *Sphingomonas* | 7.2 | 11.4 | 25.6 | 0.3 | 0.0 | 0.0 | 14.2 | 2.4 |
| *Rhizobium* | 9.6 | 23.8 | 6.3 | 4.0 | 2.5 | 0.0 | 8.1 | 0.0 |
| *Hymenobacter* | 8.6 | 3.3 | 10.0 | 7.9 | 8.9 | 7.9 | 9.9 | 7.0 |
| *Caulobacter* | 5.7 | 27.8 | 31.5 | 0.0 | 0.0 | 0.0 | 10.8 | 0.0 |
| *Levilinea* | 10.8 | 11.7 | 11.3 | 11.0 | 12.4 | 11.7 | 12.8 | 10.6 |
| *Bacillus* | 18.4 | 2.5 | 27.1 | 0.6 | 0.9 | 0.6 | 32.3 | 2.3 |
| *Clostridium* | 7.8 | 10.4 | 31.3 | 0.5 | 0.6 | 0.5 | 33.9 | 0.5 |
| *Methylobacterium* | 5.4 | 39.8 | 9.7 | 0.3 | 0.5 | 0.2 | 16.3 | 0.0 |
| *Desulfovibrio* | 11.3 | 13.0 | 14.2 | 7.4 | 8.0 | 7.0 | 11.3 | 6.2 |
| *Anabaena* | 12.2 | 5.4 | 10.2 | 13.4 | 15.1 | 13.5 | 12.9 | 13.0 |
| *Bradyrhizobium* | 1.2 | 122.3 | 7.4 | 0.6 | 0.9 | 0.6 | 5.2 | 0.5 |
| *Rhodopseudomonas* | 0.0 | 26.6 | 24.5 | 0.0 | 0.0 | 0.0 | 14.9 | 0.0 |
| *Leuconostoc* | 5.7 | 0.0 | 9.0 | 3.9 | 5.5 | 0.0 | 8.0 | 0.4 |
| *Candidatus Liberibacter* | 5.4 | 27.7 | 19.7 | 0.0 | 0.0 | 0.0 | 20.2 | 0.0 |
| *Dietzia* | 7.6 | 7.2 | 8.4 | 0.0 | 0.9 | 0.0 | 9.8 | 0.3 |

S1 Table 2b: Fluorescence data (group 2) for the most abundant probes at the genera level

| Genera | SVN40 | SVN44 | SVN48 | SVN52 | SVN56 | SVN60 |
| --- | --- | --- | --- | --- | --- | --- |
| *Brevundimonas* | 10.4 | 38.4 | 35.7 | 25.3 | 12.8 | 18.6 |
| *Moorella* | 12.4 | 16.9 | 15.5 | 16.7 | 0.0 | 12.4 |
| *Pseudomonas* | 4.3 | 6.7 | 5.3 | 6.9 | 4.1 | 5.5 |
| *Mesorhizobium* | 7.5 | 10.2 | 8.4 | 19.0 | 8.0 | 7.3 |
| *Sulfitobacter* | 9.9 | 11.6 | 11.0 | 11.3 | 9.3 | 9.8 |
| *Sphingomonas* | 1.1 | 21.1 | 0.5 | 11.7 | 3.5 | 0.6 |
| *Rhizobium* | 5.6 | 11.7 | 8.2 | 9.5 | 6.9 | 6.0 |
| *Hymenobacter* | 12.6 | 9.1 | 8.4 | 12.7 | 6.3 | 7.4 |
| *Caulobacter* | 2.2 | 14.5 | 5.7 | 19.7 | 5.8 | 3.4 |
| *Levilinea* | 4.9 | 7.2 | 6.2 | 9.1 | 2.1 | 3.6 |
| *Bacillus* | 9.4 | 11.9 | 1.6 | 5.0 | 0.4 | 3.1 |
| *Clostridium* | 0.8 | 7.5 | 0.8 | 2.4 | 0.9 | 0.8 |
| *Methylobacterium* | 1.2 | 18.3 | 5.2 | 5.1 | 4.9 | 5.3 |
| *Desulfovibrio* | 3.8 | 9.2 | 0.0 | 10.1 | 0.0 | 4.5 |
| *Anabaena* | 7.5 | 4.2 | 3.4 | 6.3 | 2.4 | 3.1 |
| *Bradyrhizobium* | 0.4 | 0.9 | 0.4 | 0.8 | 0.4 | 1.7 |
| *Rhodopseudomonas* | 0.0 | 14.5 | 0.0 | 21.6 | 0.0 | 0.0 |
| *Leuconostoc* | 12.2 | 7.2 | 5.9 | 12.1 | 5.2 | 5.9 |
| *Candidatus Liberibacter* | 0.0 | 6.1 | 0.0 | 19.1 | 0.0 | 0.0 |
| *Dietzia* | 3.1 | 7.4 | 6.6 | 6.4 | 4.5 | 7.2 |

S1 Table 2c: Fluorescence data (group 3) for the most abundant probes at the genera level

| Genera | SVN14 | SVN70 | SVN1 | SVN67 | SVN18 | SVN19 | SVN41 |
| --- | --- | --- | --- | --- | --- | --- | --- |
| *Brevundimonas* | 13.2 | 21.6 | 41.2 | 4.5 | 21.2 | 23.9 | 58.7 |
| *Moorella* | 13.6 | 18.4 | 19.9 | 18.6 | 14.0 | 15.2 | 15.3 |
| *Pseudomonas* | 5.9 | 10.7 | 13.0 | 8.3 | 5.6 | 7.7 | 7.3 |
| *Mesorhizobium* | 11.3 | 10.1 | 33.6 | 7.9 | 9.5 | 13.9 | 15.1 |
| *Sulfitobacter* | 11.2 | 14.0 | 13.1 | 13.5 | 11.0 | 12.4 | 11.1 |
| *Sphingomonas* | 11.6 | 10.0 | 33.0 | 0.6 | 9.1 | 15.9 | 17.5 |
| *Rhizobium* | 11.1 | 5.7 | 23.8 | 7.8 | 9.4 | 13.1 | 9.1 |
| *Hymenobacter* | 10.4 | 9.7 | 13.7 | 10.4 | 10.8 | 10.9 | 9.5 |
| *Caulobacter* | 5.7 | 7.7 | 32.4 | 0.3 | 4.0 | 6.2 | 12.2 |
| *Levilinea* | 5.3 | 11.0 | 11.8 | 13.2 | 4.3 | 10.7 | 10.8 |
| *Bacillus* | 6.7 | 9.5 | 31.5 | 1.6 | 5.5 | 39.6 | 8.0 |
| *Clostridium* | 6.2 | 5.4 | 34.1 | 0.8 | 3.0 | 26.4 | 1.6 |
| *Methylobacterium* | 9.8 | 1.2 | 39.1 | 3.0 | 6.5 | 13.5 | 4.9 |
| *Desulfovibrio* | 5.5 | 12.1 | 18.0 | 8.7 | 5.0 | 14.0 | 8.9 |
| *Anabaena* | 2.9 | 16.5 | 9.2 | 16.5 | 3.3 | 2.6 | 11.0 |
| *Bradyrhizobium* | 0.7 | 2.0 | 2.0 | 1.2 | 0.5 | 52.3 | 0.7 |
| *Rhodopseudomonas* | 0.0 | 9.7 | 25.1 | 0.0 | 0.0 | 26.5 | 12.4 |
| *Leuconostoc* | 9.6 | 6.4 | 13.2 | 7.4 | 9.7 | 13.3 | 8.3 |
| *Candidatus Liberibacter* | 0.0 | 19.6 | 23.9 | 0.0 | 0.0 | 5.7 | 6.0 |
| *Dietzia* | 5.1 | 9.4 | 9.1 | 1.1 | 5.3 | 6.4 | 6.7 |

S1 Table 2d: Fluorescence data (groups 4, 5, 6) for the most abundant probes at the genera level

|  | Group 4 | | | | | Group 5 | Group 6 | | |
| --- | --- | --- | --- | --- | --- | --- | --- | --- | --- |
| Genera | SVN8 | SVN23 | SVN35 | SVN66 | SVN72 | SVN49 | SVN7 | SVN24 | SVN36 |
| *Brevundimonas* | 29.5 | 32.1 | 25.9 | 18.2 | 34.0 | 0.0 | 62.9 | 30.0 | 17.4 |
| *Moorella* | 16.1 | 15.9 | 15.0 | 17.8 | 18.1 | 0.0 | 20.3 | 15.7 | 0.0 |
| *Pseudomonas* | 7.3 | 6.7 | 9.2 | 9.9 | 49.7 | 5.1 | 51.2 | 6.7 | 6.0 |
| *Mesorhizobium* | 16.5 | 23.3 | 9.5 | 9.1 | 11.5 | 0.0 | 41.6 | 11.3 | 7.2 |
| *Sulfitobacter* | 12.3 | 11.1 | 11.3 | 13.2 | 12.9 | 11.6 | 13.0 | 11.9 | 11.4 |
| *Sphingomonas* | 14.4 | 23.3 | 3.7 | 0.5 | 21.3 | 0.0 | 47.9 | 1.2 | 0.2 |
| *Rhizobium* | 11.6 | 12.5 | 8.8 | 7.1 | 14.0 | 0.0 | 26.3 | 11.5 | 6.3 |
| *Hymenobacter* | 11.3 | 12.9 | 9.9 | 10.4 | 9.4 | 0.0 | 14.5 | 5.9 | 7.3 |
| *Caulobacter* | 11.1 | 11.2 | 9.6 | 3.4 | 9.3 | 0.0 | 21.9 | 7.3 | 3.0 |
| *Levilinea* | 9.1 | 6.3 | 8.3 | 12.2 | 8.2 | 9.5 | 11.7 | 8.8 | 10.7 |
| *Bacillus* | 6.4 | 9.6 | 7.2 | 5.4 | 7.1 | 0.5 | 22.9 | 5.0 | 0.7 |
| *Clostridium* | 7.6 | 4.5 | 5.5 | 0.7 | 8.1 | 0.0 | 29.5 | 8.8 | 2.1 |
| *Methylobacterium* | 4.2 | 12.1 | 4.6 | 5.0 | 6.6 | 0.5 | 18.0 | 6.1 | 3.6 |
| *Desulfovibrio* | 5.3 | 5.4 | 6.4 | 10.6 | 6.6 | 0.0 | 14.4 | 7.2 | 6.6 |
| *Anabaena* | 3.4 | 3.3 | 3.9 | 15.5 | 3.7 | 5.0 | 8.7 | 3.9 | 11.2 |
| *Bradyrhizobium* | 0.7 | 0.9 | 1.2 | 2.3 | 1.3 | 0.5 | 56.8 | 0.6 | 0.6 |
| *Rhodopseudomonas* | 20.6 | 0.0 | 17.5 | 0.0 | 0.0 | 0.0 | 29.6 | 0.0 | 0.0 |
| *Leuconostoc* | 10.4 | 11.5 | 9.0 | 8.4 | 6.9 | 0.0 | 14.2 | 0.0 | 2.0 |
| *Candidatus Liberibacter* | 3.3 | 5.4 | 4.4 | 0.0 | 4.2 | 0.0 | 25.0 | 5.8 | 0.0 |
| *Dietzia* | 7.6 | 6.9 | 11.1 | 4.8 | 7.9 | 0.0 | 9.3 | 8.6 | 7.5 |

S1 Table 2e: Fluorescence data (groups 7, 8) for the most abundant probes at the genera level

|  | Group 7 | | | | Group 8 | |  |  |  |
| --- | --- | --- | --- | --- | --- | --- | --- | --- | --- |
| Genera | SVN64 | SVN69 | SVN68 | SVN71 | SVN65 | SVE1 | SVE2 | SVE4 | SVE7 |
| *Brevundimonas* | 23.6 | 54.6 | 27.9 | 34.2 | 26.4 | 16.4 | 10.6 | 7.7 | 12.1 |
| *Moorella* | 15.3 | 16.4 | 14.2 | 13.9 | 16.8 | 15.1 | 18.9 | 12.9 | 15.4 |
| *Pseudomonas* | 7.9 | 40.6 | 5.0 | 12.6 | 7.5 | 6.9 | 10.2 | 6.3 | 37.3 |
| *Mesorhizobium* | 9.9 | 31.0 | 16.9 | 9.7 | 7.8 | 9.6 | 9.8 | 8.6 | 10.1 |
| *Sulfitobacter* | 11.7 | 12.5 | 10.8 | 11.2 | 12.1 | 11.4 | 13.8 | 11.2 | 12.1 |
| *Sphingomonas* | 3.6 | 25.5 | 12.6 | 16.4 | 4.4 | 5.4 | 5.1 | 0.4 | 8.4 |
| *Rhizobium* | 10.8 | 11.4 | 11.4 | 11.9 | 6.1 | 8.8 | 5.0 | 7.7 | 9.3 |
| *Hymenobacter* | 9.7 | 11.3 | 11.0 | 7.5 | 8.3 | 6.3 | 7.5 | 4.9 | 7.6 |
| *Caulobacter* | 6.1 | 26.2 | 5.4 | 9.3 | 6.9 | 5.7 | 6.7 | 4.3 | 6.1 |
| *Levilinea* | 7.8 | 9.6 | 5.7 | 6.5 | 10.4 | 6.0 | 10.3 | 6.6 | 6.8 |
| *Bacillus* | 2.1 | 16.3 | 5.3 | 2.1 | 3.2 | 4.2 | 9.8 | 6.3 | 3.8 |
| *Clostridium* | 5.8 | 17.7 | 2.6 | 2.7 | 9.8 | 6.3 | 16.5 | 14.8 | 7.8 |
| *Methylobacterium* | 4.5 | 18.2 | 6.2 | 9.0 | 1.0 | 0.9 | 1.2 | 1.0 | 2.9 |
| *Desulfovibrio* | 6.7 | 8.8 | 0.0 | 6.2 | 5.8 | 3.7 | 11.1 | 5.7 | 6.1 |
| *Anabaena* | 3.9 | 12.7 | 2.5 | 2.6 | 12.2 | 3.0 | 4.3 | 3.1 | 3.3 |
| *Bradyrhizobium* | 0.5 | 1.2 | 0.3 | 0.4 | 1.0 | 0.4 | 4.7 | 0.5 | 0.5 |
| *Rhodopseudomonas* | 0.0 | 16.1 | 0.0 | 0.0 | 0.0 | 0.0 | 13.1 | 0.0 | 0.0 |
| *Leuconostoc* | 9.6 | 10.8 | 9.9 | 5.8 | 4.9 | 0.0 | 4.4 | 0.0 | 4.3 |
| *Candidatus Liberibacter* | 0.0 | 24.7 | 0.0 | 4.4 | 0.0 | 0.0 | 15.3 | 0.0 | 0.0 |
| *Dietzia* | 7.6 | 7.1 | 5.0 | 5.6 | 7.5 | 5.6 | 9.3 | 7.3 | 6.8 |

S1 Table 3: Positive hybridizations for different bacterial classes as a function of phyla/class. Values are given as averaged counts and percent of total per group.

|  | |  | 1 | | 2 | | 3 | | 4 | | 5 | | 6 | | 7 | | 8 | |
| --- | --- | --- | --- | --- | --- | --- | --- | --- | --- | --- | --- | --- | --- | --- | --- | --- | --- | --- |
| Class | Total probe count | | counts | % | counts | % | counts | % | counts | % | counts | % | counts | % | Counts | % | counts | % |
| *Alpha Proteobacteria* | 6000 | | 233.0 | 3.9 | 233.3 | 3.9 | 292.6 | 4.9 | 265.0 | 4.4 | 15.0 | 0.3 | 397.3 | 6.6 | 373.3 | 6.2 | 152.6 | 2.5 |
| *Gamma Proteobacteria* | 4904 | | 235.6 | 4.8 | 159.0 | 3.2 | 272.1 | 5.5 | 230.0 | 4.7 | 82.0 | 1.7 | 366.0 | 7.5 | 200.8 | 4.1 | 248.6 | 5.1 |
| *Beta Proteobacteria* | 1838 | | 24.3 | 1.3 | 20.5 | 1.1 | 39.1 | 2.1 | 16.6 | 0.9 | 3.0 | 0.2 | 62.3 | 3.4 | 40.3 | 2.2 | 52.2 | 2.8 |
| *Delta Proteobacteria* | 590 | | 15.2 | 2.6 | 7.7 | 1.3 | 20.0 | 3.4 | 14.4 | 2.4 | 1.0 | 0.2 | 22.0 | 3.7 | 11.0 | 1.9 | 11.4 | 1.9 |
| *Actinobacteria* | 3576 | | 326.4 | 9.1 | 241.0 | 6.7 | 412.5 | 11.5 | 258.0 | 7.2 | 12.0 | 0.3 | 520.7 | 14.6 | 333.5 | 9.3 | 217.8 | 6.1 |
| *Firmicutes* | 5164 | | 224.5 | 4.3 | 141.2 | 2.7 | 275.8 | 5.3 | 146.2 | 2.8 | 6.0 | 0.1 | 224.0 | 4.3 | 146.0 | 2.8 | 88.4 | 1.7 |
| *Cyanobacteria* | 1588 | | 128.6 | 8.1 | 182.7 | 11.5 | 208.7 | 13.1 | 143.6 | 9.0 | 16.0 | 1.0 | 208.7 | 13.1 | 165.5 | 10.4 | 87.4 | 5.5 |
| *Bacteroidetes* | 1715 | | 52.6 | 3.1 | 35.7 | 2.1 | 53.8 | 3.1 | 61.6 | 3.6 | 6.0 | 0.3 | 79.7 | 4.6 | 60.3 | 3.5 | 47.4 | 2.8 |
| *Spirochaetes* | 319 | | 27.1 | 8.5 | 18.3 | 5.7 | 19.5 | 6.1 | 0.4 | 0.1 | 0.0 | 0.0 | 36.7 | 11.5 | 0.0 | 0.0 | 0.0 | 0.0 |
| *Crenarchaeota* | 92 | | 4.6 | 5.0 | 6.0 | 6.5 | 6.3 | 6.9 | 6.2 | 6.7 | 0.0 | 0.0 | 10.7 | 11.6 | 6.0 | 6.5 | 6.2 | 6.7 |
| *Chlorobi* | 28 | | 6.0 | 21.4 | 6.2 | 22.0 | 6.0 | 21.4 | 6.0 | 21.4 | 0.0 | 0.0 | 6.0 | 21.4 | 6.0 | 21.4 | 6.0 | 21.4 |
| *Acidobacteria* | 45 | | 5.6 | 12.5 | 6.0 | 13.3 | 6.2 | 13.7 | 7.0 | 15.6 | 0.0 | 0.0 | 5.0 | 11.1 | 7.3 | 16.1 | 7.0 | 15.6 |
| *Euryarchaeota* | 371 | | 6.3 | 1.7 | 3.7 | 1.0 | 9.8 | 2.7 | 8.0 | 2.2 | 2.0 | 0.5 | 8.3 | 2.2 | 1.5 | 0.4 | 1.8 | 0.5 |
| *Planctomycetes* | 170 | | 6.0 | 3.5 | 1.8 | 1.1 | 7.5 | 4.4 | 3.8 | 2.2 | 2.0 | 1.2 | 16.7 | 9.8 | 2.5 | 1.5 | 2.0 | 1.2 |
| *Chloroflexi* | 78 | | 5.3 | 6.7 | 2.8 | 3.6 | 4.2 | 5.3 | 3.4 | 4.4 | 2.0 | 2.6 | 5.3 | 6.8 | 6.0 | 7.7 | 3.4 | 4.4 |
